# Comprehensive analysis of carbon emissions, economic growth, and employment from the perspective of industrial restructuring: a case study of China

**DOI:** 10.1007/s11356-021-14040-z

**Published:** 2021-05-10

**Authors:** Shukuan Bai, Boya Zhang, Yadong Ning, Ying Wang

**Affiliations:** Key Laboratory of Ocean Energy Utilization and Energy Conservation of Ministry of Education, School of Energy and Power Engineering, Dalian University of Technology, No.2 Linggong Road, High-Tech Pack, Dalian, 116024 Liaoning China

**Keywords:** Low-carbon transition, Industrial restructuring, Employment impacts, Semi-closed input–output model, Households, Partially endogenized consumption, China

## Abstract

Industrial restructuring is a significant measure for low-carbon transition. In principle, carbon emissions can be effectively reduced by limiting the output of high-emission sectors; however, the socio-economic effects of the sectors should also be considered. Moreover, owing to the limitations of the method or data, the interactions between households and production sectors have been neglected in the study of industrial restructuring, resulting in an incomplete and potentially biased understanding of the role of households. To fill this gap, we applied a semi-closed input–output model to identify key sectors by economic and emission linkages and measure the employment impacts (direct, indirect, and induced) of reduced carbon emissions. The empirical results for China in 2010–2018 showed that relatively small changes in key emission sectors would significantly affect the economic growth, and reduced carbon emissions reduction would generally lead to high job losses. Promoting labor-intensive sectors, particularly the service sector, is conducive to achieving a “multi-win” situation for economic development, carbon emission reductions, and stable employment. Furthermore, our results highlight the significance of households: expanding consumption and increasing household income can bring multiple benefits, such as economic growth, job creation, and low carbon emissions. These findings can provide useful information for identifying the optimized path of restructuring and helping achieve the sustainable development of the environment, economy, and society.

## Introduction

With the rapid development of the world economy, energy supply and environmental problems have become increasingly severe (IPCC 2013; Quadrelli and Peterson 2007). The transition to a low-carbon economy has become a basic strategy for countries worldwide to help address climate change, maintain energy security, and reduce environmental pollution (de Miguel et al. 2019). As the world’s largest developing economy, China has achieved remarkable socio-economic progress at large resource and environmental costs since the reform and opening-up about 40 years ago. China has become the world’s largest emitter of CO_2_, with CO_2_ emissions from fuel combustion increasing to 9570.8 Mt in 2018, accounting for 28.6% of the world’s total (IEA 2020). Therefore, accelerating the transition to a low-carbon economy is not only imperative for China’s sustainable development but also a strategic choice to actively engage in the global climate change campaign. Industrial restructuring and optimization are important means for achieving economic transition and reduced carbon emissions (Wen and Wang 2019).

Industrial restructuring is a phenomenon that adjusts the proportions of various sectors to meet one or more target (Mi et al. 2015). Without considering other targets, CO_2_ emissions can be effectively reduced by restricting high-emission sectors but expanding low-emission sectors. However, in practice, reducing emissions by output restriction will influence the economic income of some sectors and may hinder national economic growth to some extent. Consequently, economic and emission performance should be evaluated jointly when discussing the roles of various sectors in a country (Chang 2015). Further, employment impacts are one of the most important social impacts associated with industrial restructuring, as well as one of the most crucial concerns for formulating policies. The adjustment of industrial structures will inevitably affect employment (industry is an important carrier of labor) and China’s large pool of human resources. Given the increasing employment pressure and permanent structural contradictions, employment impacts should be considered during industrial restructuring. Protecting the environment, promoting the economy, and maintaining social stability are three important dimensions for sustainable development. Achieving a multi-win situation for the environment, economy, and society has aroused widespread attention from all sectors of society and is the main motivation for conducting this study.

In addition to considering multiple targets, industrial restructuring needs to focus on sectoral linkages. Given that sectors are closely linked in an economy and there are complex input–output linkages between sectors, measures implemented in a sector could significantly affect other sectors. For instance, some emission-intensive sectors involve longer industrial chains and occupy crucial positions in the supply chain as prominent suppliers of raw materials and intermediate products to other sectors. Limiting the output of these sectors will not only constrain the sector itself, but it can also affect the development of related sectors through complicated sectoral linkages and even the entire production chain. Consequently, industrial restructuring should not only focus on the role of individual sectors but also the intersectoral linkages. Linkage analysis is an effective approach that enables us to look deep into the intersectoral linkages and identify the direction of industrial restructuring for a low-carbon economy (Chang 2015). Comparisons of the strength of linkages for the sectors in an economy provide one mechanism for identifying key sectors in that economy—those sectors that are most connected and, therefore, in some sense, most “important” (Miller and Blair 2009).

Industrial restructuring typically concerns the structure of production sectors; however, households also play an important role in promoting economic and social operations, and the interactions between households and production sectors require attention. Household labor remuneration has a ripple effect on the production of various industrial sectors, while household consumption acts as an important link in the labor reproduction chain, as well as the fundamental basis and condition of economic activities. Consequently, households affect the output of production sectors through household consumption and further affect carbon emissions; in turn, production sectors affect household consumption spending through labor compensation (Zhang et al. 2017). Furthermore, for the analysis of social impacts, changes in the income and consumption of the workers employed in a particular set of related industries will induce considerable employment changes. Therefore, the induced employment impact resulting from the changes in household consumption and labor income requires attention (Markaki et al. 2013). Unfortunately, owing to the uncertainty and limitations of the method or data, the production–household linkage and induced employment impact have usually been neglected or poorly measured, leading to an incomplete and potentially biased understanding of the role of households and their social impacts.

Against this background, this paper aims to provide an input–output based framework to identify the direction of industrial restructuring for a low-carbon transition by economic and emission linkage analysis and to quantify the full scope of the employment impacts of carbon emissions reduction (direct, indirect, and induced). A semi-closed input–output model with partially endogenized consumption is used to evaluate sectoral linkages and quantify employment impacts for the first time. Unlike the conventional input–output analysis, this model considers both the production–household linkage and household consumption structure (endogenous and exogenous). On this basis, this study calculated the economic and carbon emission diffusion coefficients of 28 sectors in China and analyzed the economic and emission linkages to identify key sectors of industrial restructuring. In this study, we conducted a full-scope evaluation of the employment impacts—particularly the induced effects—and showed reduced deviations in the evaluation results. More comprehensive interactions between production sectors and households can be observed by these means, and our method will help reduce deviations when measuring true impacts and provide useful information for making policies. This study proposes a new indicator, job losses per unit of carbon emissions reduction, which makes it possible to intuitively quantify the impacts of reduced carbon emissions on employment and may further contribute to reducing the negative impacts of carbon emission reduction measures on the labor market.

The remainder of this paper is organized as follows. The “Literature review” section reviews the existing literature on sectoral linkage analysis and employment impacts, the “Methodology and data” section describes the model and data employed in this research, the “Results and discussion” section presents the main results and a detailed discussion, and the “Conclusions and policy implications” section provides the conclusions and policy implications.

## Literature review

Sectoral linkage analysis is one of the common methods in the study of industrial restructuring. One well-known early study that is often cited in research on linkage analysis is that of Chenery and Watanabe (1958), who used linkages to analyze the structure of production; their report brought an increasing amount of literature on this topic (Jones 1976; Cella 1984; Dietzenbacher 1992; Dietzenbacher and Van Der Linden 1997; Luo 2013). Recently, linkage analysis methods have been applied to address environmental issues, particularly energy and carbon emissions (Guo et al. 2018; Wen and Wang 2019; Wang et al. 2020). In the literature on linkage analysis, backward and forward linkages are concepts usually used to describe the relationships between sectors (Cai and Leung 2004). Generally, backward linkages are the case wherein a sector’s final demand can pull other sectors’ output (Zhao et al. 2015). According to the classical input–output theory, the final demand is the driving force of economic growth, and backward linkage is important when determining CO_2_ emissions driven by the final demand for goods and services (e.g., household consumption, government consumption, and capital accumulation) in the supply chain (Peters 2008; Wang et al. 2017; Ma et al. 2019). Meanwhile, forward linkages are the case wherein additional supply from a sector will push other sectors to use the additional output and produce more products. The Ghosh model is often used to measure the forward linkage from the supply perspective (Dietzenbacher 2002). Owing to the joint stability problem of input–output production and allocation coefficients (Chen and Rose 1989; Dietzenbacher 1989), there are some debates on the simultaneous use of the Leontief and Ghosh models (Cella 1984; Lenzen 2003). For the purposes of our study, it is advisable to conduct linkage analysis from the demand perspective compared to the supply-driven Gosh model. Therefore, this paper mainly focuses on backward linkages based on the Leontief model.

Numerous studies have conducted sectoral linkage analysis for identifying key sectors. Wen and Wang (2019) found that construction was China’s crucial sector in the backward direction. Guo et al. (2018) found that while driving energy consumption and carbon emissions in other sectors, China’s key sectors consume large amounts of fossil energy and generate large amounts of emissions owing to the demands of other sectors. The results reported by Wang et al. (2020) showed that, among all the backward linkage-related key sectors, the production and supply of electricity and water have the greatest pulling effects, followed by metal mining. Although these impressive works have inspired our study, two points require further discussion. First, carbon emission reductions and economic growth should be considered jointly when identifying key sectors for a developing economy. Most studies on sectoral linkage analysis focus on only the monetary (Chenery and Watanabe 1958; Jones 1976; Cella 1984; Dietzenbacher 1992; Dietzenbacher and Van Der Linden 1997; Luo 2013) or environmental issues (Guo et al. 2018; Zhao et al. 2015). As mentioned above, adjusting the industrial structure to meet carbon emission reduction targets will limit the development of some industries, which may damage the national economy (Chang 2015; Wen and Wang 2019). Owing to the increasing pressing environmental issues, it is of practical significance to consider economic growth in the issue of carbon emissions reduction to identify key sectors with above-average economic performance and below-average environmental performance (Lenzen 2003; Wang et al. 2013b). Chang (2015) found that expanding the concept of production linkage into emission issues resulted in considerably different consequences. Therefore, to balance the targets of economic growth and carbon emission reductions, the economic and carbon emissions linkages are analyzed jointly in this study to evaluate the role of various sectors and identify key sectors of industrial restructuring.

Second, within the input–output framework, previous sectoral linkage analysis (Jones 1976; Cella 1984; Dietzenbacher 1992; Dietzenbacher and Van Der Linden 1997; Luo 2013; Guo et al. 2018; Wang et al. 2020) mainly uses the traditional open input–output models, which considers the household consumption an exogenous variable of the model, while production sectors are considered endogenous. Zhang et al. (2017) pointed out that previous studies have neglected the fact that the traditional input–output model can only capture the impact of households on production sectors. In practice, household consumption and production activities interact and affect each other, resulting in two-way feedback between production sectors and households. Production sectors affect household consumption spending through labor compensation, and, in turn, household consumption affects carbon emissions and the output of production sectors (Zhang et al. 2017). To address this gap, the semi-closed input–output model provides the possibility of considering the production–household linkage in corresponding situations (Chen et al. 2015). Because of the model’s advantage in dealing with household consumption and income, it has attracted considerable attention from researchers. Miyazawa’s (1976) important study introduced the distribution of income and expenditure into the input–output model and led to further studies by Batey (1985); Cloutier and Thomasin (1994); Sonis and Hewings (1999); Wakabayashi and Hewings (2007), and Steenge et al. (2020). These studies have been focused on dividing the households into different groups by certain characteristics and made a valuable contribution concerning income distribution questions and demographic economic analysis. However, one limitation of the semi-closed input–output model has received scant attention. Chen et al. (2016) indicated that this model does not accurately reflect real household consumption behavior, because household consumption is fully endogenized and neglects the household consumption structure in this model. More specifically, the model assumes that current consumption depends only on current income (Dietzenbacher and Günlük-Şenesen 2003). However, household consumption is also affected by several other factors, such as previous spending levels, expected revenue, and consumption habits, according to the relative income hypothesis and the life cycle-permanent income hypothesis (Friedman 1957; Modigliani 1986). The role of households is exaggerated in fully endogenized consumption models. To address this problem, a semi-closed input–output model with partially endogenized consumption was proposed and applied to examine the impacts of the Chinese stimulus package on the gross domestic product (Chen et al. 2016). Following Chen’s framework, linkage indicators based on the semi-closed input–output model with partially endogenized consumption were conducted in this study.

As one of the most significant social impacts associated with industrial restructuring, employment impacts have received increasing attention (Mu et al. 2018). Recently, there has been an increasing amount of literature on employment impacts. Generally, employment impacts tend to be summarized into three categories, namely, direct, indirect, and induced, based on the relevancy of economic impacts (Lambert and Silva 2012). As an illustration of the three different effects, consider a sector *j*: if there is a unit increase in final demand for a product of sector *j*, it can be assumed that there will be an equivalent increase in the output of that sector as producers react to meet the increased demand. Employment will be generated in that sector as a result of the new output, which is the direct effect. As the output of sector *j* increases, there will also be an increase in the demand on its domestic suppliers and so on in the supply chain, and employment will also increase in these sectors, which is the indirect effect. Owing to these direct and indirect increases in employment, the household income level will grow across the domestic economy. A proportion of this increased income will be re-spent on domestically produced products, generating employment in each sector based on their newly increased outputs, which is the induced effect (Miller and Blair 2009).

In recent years, two primary approaches have been attempted to investigate employment impacts, analytical and input–output methods, both of which have their advantages and drawbacks. Analytical methods are commonly utilized to evaluating the employment impact at a regional or local level, which generally relies on extensive surveys. For instance, Moreno and López (2008) conducted an analytical study of forecasting employment generated by renewable energy, Wei et al. (2010) presented an analytical model used for estimating net employment effects based on different policies and scenarios, and Yi (2013) estimated the number of green jobs for climate and clean energy policies. The analytical method tends to be more transparent, easily understood, and able to have its sensitivity evaluated; however, it often provides direct effects and may not be able to capture the indirect and induced effects (Blanco and Rodrigues 2009; Lambert and Silva 2012). Meanwhile, input–output methods are the most well-known tools for analyzing the impacts of a sector on all the other sectors in an economy and are typically used for determining the economic impacts of a particular investment or activity. In recent years, input–output methods have been increasingly applied to study the countrywide employment impacts arising from changes in a particular sector. Such studies involve the employment impacts of the power (Stoddard et al. 2006; Tourkolias and Mirasgedis 2011), wind energy (Ulrich et al. 2012; Simas and Pacca 2014), biofuel (Malik et al. 2014), and renewable energy sectors (Garrett-Peltier 2017; O’Sullivan and Edler 2020). When evaluating employment impacts, the input–output method often adopts one of the following models: the input–output model, social accounting matrices, computable general equilibrium models, and other extensions of input–output models (Alarcon et al. 2011; Lambert and Silva 2012; Markaki et al. 2013; Zhou 2017). Compared to the analytical methods, input–output methods can determine direct and indirect effects and enable the calculation of induced effects caused by changes in labor income and consumption in certain models (Bohlmann et al. 2019).

Over the past decade, researchers have shown an increased interest in evaluating the employment impacts in China. A case study of China’s power generation sector by Cai et al. (2011) found that, when evaluating the employment impacts, it is necessary to consider not only the intuitive direct impacts but also the indirect impacts, which may have a greater effect on final results. Wang et al. (2013a) evaluated the employment impacts of renewable energy policies in China and calculated both the direct and indirect employment impacts of the clean development mechanism projects on the power sector. Their results showed that employment impacts are mainly manifested in the employment opportunities created by other sectors. Mu et al. (2018) found that induced employment impacts have usually been neglected, resulting in an incomplete result. To date, far too little attention has been paid to the induced effects for the limitations of the method or data, which may lead to downward deviation of quantification of employment impacts. However, all effects should be considered to obtain a comprehensive and accurate evaluation of the employment impacts. Previous studies have failed to fully consider the induced effects. Although these effects can be evaluated using the traditional semi-closed input–output model, the linkage between the household and production sectors will most likely be overestimated. Consequently, results regarding the employment impacts obtained from this model will most probably be biased in an upward sense (Chen et al. 2016). This happens because, as mentioned previously, the traditional semi-closed input–output model is a fully endogenized consumption model, the household consumption structure (endogenous and exogenous) has been neglected. Therefore, a semi-closed input–output model with partially endogenized consumption was adopted by this study to reevaluate the employment impacts. It is conducive to more accurately measure the impact of household consumption and income on employment. Based on conventional employment multipliers, this study established a new indicator (job losses per unit of carbon emissions reduction) to intuitively quantify the employment impacts of carbon emissions reduction, which may contribute to reducing the shock to labor employment caused by carbon emissions reduction policies. Thus, it is of great practical significance for both the low-carbon transition and maintenance of employment levels in China.

## Methodology and data

As a well-structured model, the input–output model can evaluate the economy-wide and sectoral impacts of the changes in the final demand for outputs of a particular sector or group of sectors on key socio-economic variables, such as output, value added, and employment. The input–output model can be categorized into three types, namely, open, closed, and semi-closed, based on its method of processing the final demand. Given that households play an active part in driving production and maintaining social operation, the interactions between households and production sectors cannot be neglected. This study adopted a semi-closed input–output model to capture the interactions between households and production sectors. The basic assumption of the input–output model is valid in this study.

### Semi-closed input–output model with partially endogenized consumption

In the conventional semi-closed input–output model, households are fully endogenized. The row vector (labor input row) is the labor remuneration paid by each sector and the income distributed to households through other means, whereas the column vector is the consumption of various sectors’ products and services by households. In the semi-closed input–output model with partially endogenized consumption, a consumption decomposition formula is used to divide household consumption into endogenous and exogenous consumption, which are determined by the current household income and other factors, respectively. Accordingly, household income is also divided into exogenous and endogenous income, which are defined as income from labor compensation related to production and income not related to production in the accounting period (e.g., income from wealth and transfers), respectively (Chen et al. 2016). Only endogenous parts are closed into the intermediate flow matrix in this model, this further improves the semi-closed model in terms of its consideration of household consumption structure and can even more accurately reflect the interactions between households and production sectors. Thus, it is a good tool for impact analyses, especially at the sector level.

In the semi-closed input–output model with partially endogenized consumption, the construction process of the household column vector (endogenous consumption) mainly includes the following steps: estimating the endogenous consumption coefficient of eight categories of consumption commodities, using the estimated bridge matrix to convert the endogenous consumption coefficient of eight categories of consumption commodities to those of input–output sectors, and calculating endogenous and exogenous consumption using the endogenous consumption coefficient and consumption decomposition formula. The estimation of the endogenous consumption coefficient adopts the time-varying parameter model, maximum likelihood estimation, and Kalman filtering algorithm (Hamilton 1994; Havey 1987). The construction of the household row vector (endogenous income) mainly uses statistical data to estimate the endogenous income coefficient and then calculates the exogenous and endogenous income. A more detailed modeling process is presented in Appendix A. The framework of the semi-closed input–output model with partially endogenized consumption is shown in Table 1. Accordingly, the following balance equation can be obtained:

1 $$ \sum \limits_{j=1}^n{Z}_{ij}^d+{C}_i^{d, en}+{C}_i^{d, ex}+{C}_i^{d,g}+{F}_i^d+{E}_i^d={X}_i, $$

2 $$ \sum \limits_{j=1}^n{H}_j+{h}^{ex}={x}_{n+1}. $$

**Table 1:**
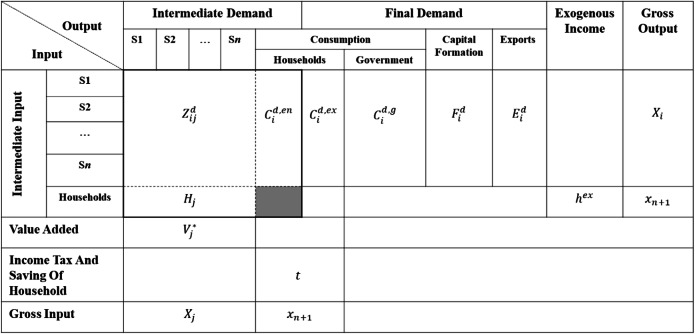
Framework of the semi-closed input–output model with partially endogenized consumption

Here ${Z}_{ij}^d$ denotes the intermediate input from sector *i* that flows to sector *j*; ${C}_i^{d, en}$ and ${C}_i^{d, ex}$, the endogenous and exogenous consumption of goods and services delivered by domestic sector *i* to households, respectively; ${C}_i^{d,g}$, the value of government purchases of goods and services from sector *i*; ${F}_i^d$, the value of gross capital formation of sector *i*; ${E}_i^d$, the export of goods and services from sector *i*; *X*_*i*_, the gross output of sector *i*; *x*_*n* + 1_, the total household income (composed of two parts: endogenous income *H*_*j*_ and exogenous income *h*^*ex*^); and *X*_*j*_, sector *j*’s gross input, is equal to the gross output of that sector.

The expanded direct input coefficient matrix of the domestic product of this model is denoted by $$ {\mathbf{A}}^{\ast \mathbf{d}}=\left[\begin{array}{cc}{\mathbf{A}}^{\mathbf{d}}& {\mathbf{C}}^{\mathbf{d}}\\ {}\mathbf{W}^{\prime }& 0\end{array}\right] $$, where **A**^**d**^ denotes the basic direct input coefficient matrix of the domestic product, which is calculated using the open input–output model. Its elements are denoted by ${a}_{ij}^d={Z}_{ij}^d/{X}_j$. The column vector **C**^**d**^ consists of the endogenous consumption coefficient of the domestic product ${\alpha}_i^d={C}_i^{d, en}/{x}_{n+1}$. **W**′ denotes a row vector consisting of the endogenous income coefficient *w*_*j*_ = *H*_*j*_/*X*_*j*_. The interaction between the household row and household column (the shaded area of Table 1) is set to zero in our framework.^1^ The total final demand column vector of domestic product is recorded as **Y**^**d**^, whose elements are denoted by ${Y}_i^d={C}_i^{d, ex}+{C}_i^{d,g}+{F}_i^d+{E}_i^d$.

Equations (1) and (2) can be expressed in matrix form as:

3 $$ \left[\begin{array}{cc}{\mathbf{A}}^{\mathbf{d}}& {\mathbf{C}}^{\mathbf{d}}\\ {}\mathbf{W}^{\prime }& 0\end{array}\right]\left[\begin{array}{c}\mathbf{X}\\ {}{x}_{n+1}\end{array}\right]+\left[\begin{array}{c}{\mathbf{Y}}^{\mathbf{d}}\\ {}{h}^{ex}\end{array}\right]=\left[\begin{array}{c}\mathbf{X}\\ {}{x}_{n+1}\end{array}\right], $$

and Eq. (3) can be further written in the form of

4 $$ {\left(\mathbf{I}-{\mathbf{A}}^{\ast \mathbf{d}}\right)}^{-1}{\mathbf{Y}}^{\ast}={\mathbf{X}}^{\ast}, $$

where $$ {\mathbf{Y}}^{\ast}=\left[\begin{array}{c}{\mathbf{Y}}^{\mathbf{d}}\\ {}{h}^{ex}\end{array}\right] $$ and $$ {\boldsymbol{X}}^{\ast}=\left[\begin{array}{c}\boldsymbol{X}\\ {}{x}_{n+1}\end{array}\right] $$. ${\overline{\boldsymbol{B}}}^{\ast}={\left(\boldsymbol{I}-{\boldsymbol{A}}^{\ast \boldsymbol{d}}\right)}^{-1}$ is known as the Leontief inverse or the total requirements matrix.

### Measurement of sectoral linkages

Linkage analysis is a well-known approach to describe intersectoral relationships and identify key sectors. Rasmussen (1956) proposed using the column sums of the Leontief inverse to measure intersectional backward linkages. The diffusion coefficient is an important parameter to let normalized backward linkages be the measures and is defined as the ratio of a sector’s backward linkage and sector-wide average level (Liu 2002). This study adopted an improved weighted average economic diffusion coefficient to assess the economic linkage and compare different industrial sectors’ pulling impact on the economy. The formula can be expressed as follows:

5 $$ {\beta}_j=\frac{\sum \limits_{i=1}^n{\overline{b}}_{ij}^{\ast }}{\sum \limits_{j=1}^n\sum \limits_{i=1}^n{\lambda}_j{\overline{b}}_{ij}^{\ast }}, $$

where ${\overline{b}}_{ij}^{\ast }$ is the element of the total requirements matrix ${\overline{\mathbf{B}}}^{\ast}$. The denominator is the weighted average of the molecular value of various sectors, representing the sector-wide average, and the weight *λ*_*j*_ is the ratio of sector *j*’s final demand to the total final demand of all sectors. Further, *β*_*j*_ > 1 indicates that a unitary increase in final demand for sector *j*’s output will generate an above-average increase in economic activity, and the opposite is indicated when *β*_*j*_ < 1.

To analyze carbon emission linkages, first, CO_2_ emissions accounting is necessary. Fossil fuel combustion and industrial production processes are the primary sources of carbon emissions in China. Further, CO_2_ emissions from municipal waste incineration are considered in this study. Based on the accounting method of the 2006 IPCC Guidelines for National Greenhouse Gas Inventories (IPCC 2006), the accounting equation of CO_2_ emission from fossil fuel combustion is expressed as follows:

6 $$ {C}_f=\sum \limits_k{E}_{jk}\times {f}_k, $$

where *C*_*f*_ is the CO_2_ emissions of industrial sectors from fossil fuel combustion, *E*_*jk*_ is the energy *k* consumptions of industrial sector *j*, and *f*_*k*_ refers to the carbon emission coefficient of energy *k*.

The CO_2_ emission from the production processes of cement, glass, lime, iron and steel, and calcium carbide are estimated using the production-based approach recommended by the IPCC. The carbon balance-based approach is used to estimate CO_2_ emissions from the iron and steel production process. The CO_2_ emissions resulting from the oxidation of carbon in fossil waste during incineration are also estimated using the method of IPCC (2006).

Define **D** = **CX**^**∗** − 1^ as the diagonal matrix of carbon intensity coefficients, whose diagonal elements *d* are the volume of CO_2_ emission per unit output of sectors. Moreover, ***C*** denotes CO_2_ emissions from the above three sources. This can also be expressed by the following equation based on the input–output model:

7 $$ \mathbf{C}={\mathbf{DX}}^{\ast}=\mathbf{D}{\overline{\mathbf{B}}}^{\ast}{\mathbf{Y}}^{\ast}. $$

Define ${\mathbf{Q}}^{\ast}=\mathbf{D}{\overline{\mathbf{B}}}^{\ast}$ as the total carbon intensity matrix. Then, the formula for calculating the diffusion coefficient of carbon emission can be obtained:

8 $$ {\gamma}_j=\frac{\sum \limits_{i=1}^n{q}_{ij}^{\ast }}{\sum \limits_{j=1}^n\sum \limits_{i=1}^n{\lambda}_j{q}_{ij}^{\ast }}, $$

where ${q}_{ij}^{\ast }$ are elements of the total carbon intensity matrix. A similar interpretation can be used for carbon emission linkages: *γ*_*j*_ > 1 indicates that a unitary increase in the final demand for sector *j*’s output will draw an above-average increase in carbon emissions and the opposite is indicated when *γ*_*j*_ < 1.

### Measurement of employment impacts

The notion of input–output multipliers is based on the difference between the initial effect of an exogenous change (final demand) and the total effect of a change (Miller and Blair 2009). Therefore, employment multipliers can be used to estimate the total impact on employment throughout the economy arising from a change in the final demand for per unit output of the industrial sector. Found from different input–output models, the total effects can be defined either as the direct and indirect effects (open model) or as the direct, indirect, and induced effects (semi-closed model with endogenized consumption). The multipliers containing direct and indirect effects are called simple multipliers. When direct, indirect, and induced effects are included, the multipliers are referred to as total multipliers.

In general, the direct effect of sector *j* can be calculated by the labor input coefficient:

9 $$ {k}_j^{de}={L}_j/{X}_j, $$

where ${k}_j^{de}$ denotes the labor input coefficient, the subscript denotes the sector, and *L*_*j*_ denotes the employment of sector *j*.

The indirect and induced effects can be calculated by employment multipliers. The simple employment multiplier refers to the sum of direct and indirect employment changes generated by the direct output changes due to a unit increase in final demand. The simple employment multiplier for sector *j* can be calculated as follows:

10 $$ {m}_j=\sum \limits_{i=1}^n{k}_j^{de}{\overline{b}}_{ij}, $$

where ${\overline{b}}_{ij}$ is the total requirements coefficient calculated by the open input–output model.

The indirect effects of sector *j* can be calculated as follows:

11 $$ {k}_j^{ie}={m}_j-{k}_j^{de}, $$

When the employment multiplier is calculated by the semi-closed input–output model with partially endogenized consumption, the additional induced effects are captured. In this case, the employment multiplier is called the total employment multiplier and can be calculated as follows:

12 $$ {\overline{m}}_j=\sum \limits_{i=1}^n{k}_i^{de}{\overline{b}}_{ij}^{\ast }. $$

Therefore, the induced effects of sector *j* can be expressed by the difference between the total employment multiplier and the simple employment multiplier:

13 $$ {k}_j^{ue}={\overline{m}}_j-{m}_j. $$

To intuitively measure the employment impacts of carbon emission reductions, the employment multiplier was then multiplied by the output per unit of carbon emission, namely, the reciprocal value of carbon intensity coefficients. The job losses per unit of carbon emissions reduction for sector *j* can be calculated as follows:

14 $$ {n}_j={\overline{m}}_j{d_j}^{-1}, $$

where *d*_*j*_ is the carbon intensity coefficients of sector *j*. Using the direct, indirect, and induced effects as a substitute variable for total employment multiplier ${\overline{m}}_j$ in Eq. (14), it is possible to analyze the path of carbon emission reduction affecting employment and the size of the impact produced on each path.

### Data

The data used in this study were mainly collected from various statistical yearbooks in China. Taking the 2017 data as an example, the sectoral input and output data are from the input–output tables of China (NBSC 2018a). The non-competitive import table was selected in this study based on the assumption that domestic products and imports are incomplete substitutes for each other. Because the production process of imported products occurs abroad, the non-competitive import table deducts imported goods from intermediate and final use. This distinction between flows of domestically produced and imported products is necessary to avoid exaggerating the impact of the final demand, particularly the production sector, with its relatively high proportion of imports. The input–output model with non-competitive imports can more objectively evaluate the actual impact of a country’s economic activities on its domestic economy and environment, which is especially true for China with its high proportion of processing trade. To construct a non-competitive input–output table, this study drew on the method outlined in Zhang (2009) for deducting imported goods from intermediate and final use. The import and export classification data of goods trade are from the United Nations Comtrade Database (SAFE 2019), and the import and export data of services trade are obtained from the balance table of “Payments of China,” published by the State Administration of Foreign Exchange (UNSD 2015). To eliminate the impact of price fluctuations across different years, the price index provided by the China Statistical Yearbook was used to deflate the input–output tables to the 2010 constant price (NBSC 2018a). Given that the number of sectors in the input–output tables of China varies over time, to maintain consistency between different years and maintain consistency among the sectors of energy consumption, this study accordingly merged the input–output table into 28 harmonized subsectors, as presented in Table 2.

**Table 2:** Subsector classification

Code	Sector
S01	Agriculture, forestry, animal husbandry, and fishing
S02	Mining and washing of coal
S03	Extraction of petroleum and natural gas
S04	Mining and processing of metal ores
S05	Mining and processing of nonmetal ores and other ores
S06	Manufacture of foods, beverage, and tobacco
S07	Manufacture of textile
S08	Manufacture of textile wearing apparel, footwear, caps, leather, fur, feather (down), and its products
S09	Processing of timber and furniture
S10	Papermaking, printing and manufacture of articles for culture, education, and sports activities
S11	Processing of petroleum, coking and processing of nuclear fuel
S12	Chemical industry
S13	Manufacture of non-metallic mineral products
S14	Smelting and pressing of metals
S15	Manufacture of metal products
S16	Manufacture of general purpose and special purpose machinery
S17	Manufacture of transport equipment
S18	Manufacture of electrical machinery and apparatus
S19	Manufacture of communication, computers, and other electronic equipment
S20	Manufacture of measuring instrument and machinery for cultural activity and office work
S21	Manufacture of artwork, other manufacturing
S22	Production and supply of electric power and heat power
S23	Production and distribution of gas
S24	Production and supply of water
S25	Construction
S26	Traffic, transport and storage, post-service
S27	Wholesale trade and retail trade, hotels and catering services
S28	Other services

Carbon emissions from sectoral energy consumption used in this study were calculated based on the energy balance table of China and the final energy consumption by sectors, both published in the China Energy Statistical Yearbook (NBSC 2018b). The final consumption for industrial raw materials and materials used for non-energy purposes is not included. There are 14 types of energy related to carbon emission calculations in this study. Relative carbon emission coefficients were obtained from the 2006 IPCC Guidelines for National Greenhouse Gas Inventories (IPCC 2006). To calculate carbon emissions from industrial production processes and municipal waste incineration, the industrial production volumes were mainly obtained from the China Industry Statistical Yearbook and the corresponding industrial product statistical data (NBSC 2018c), and the municipal waste incineration data were collected from the China Statistical Yearbook (NBSC 2018a).

The preliminary estimated employment in each subsector was calculated by dividing its total labor compensation (from China’s input–output table) by its average wage, which comes from the China Labor Statistics Yearbook (NBSC 2018d). The number of people employed in each of three industries was obtained from the China Labor Statistics Yearbook, and the final employment figure was obtained by allocating these three industries into sectors based on each sector’s ratio in the preliminary estimation. The household income and consumption were obtained from the China Statistical Yearbook (NBSC 2018a). The statistical yearbook provided the per capita income data from urban and rural households, which consist of four parts: income from wages and salaries, net business income, net income from property, and net income from transfers. The first two parts are endogenous income, while the latter two are exogenous. The national income was calculated by multiplying the per capita income by the population. The total income was obtained by adding the endogenous and exogenous incomes. Similarly, the consumption data of eight categories of consumption commodities was also calculated based on per capita consumption multiplied by the total urban and rural population. The income and consumption data above were uniformly transformed into the 2010 constant price.

## Results and discussion

### Overviews of economic growth, carbon emissions, and employment in China

**Fig. 1:**
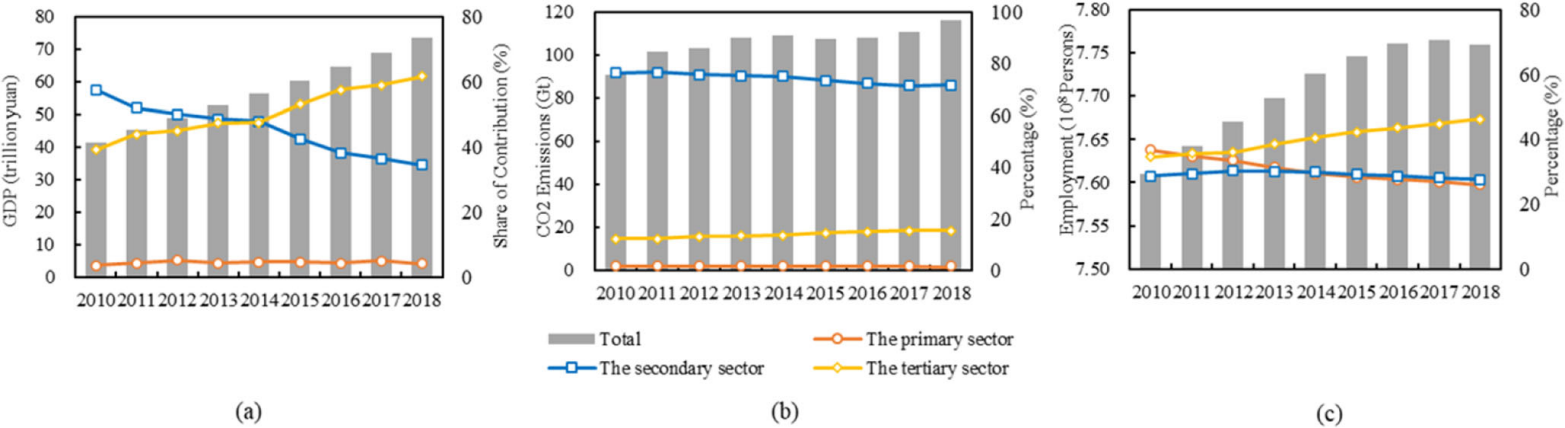
GDP (**a**), CO_2_ emission (**b**), and employment (**c**) in China by three economic sectors in 2010–2018

Figure 1 summarizes China’s historical trends in economic growth, carbon emissions, and employment from 2010 to 2018. China’s GDP and the contribution share of the three economic sectors to the increase in GDP are shown in Fig. 1a. As China became the world’s second-largest economy by 2010, its economic development entered a period of transition. During the research period, China’s GDP increased from 41.3 to 73.5 trillion yuan, but its annual growth rate slowed down. With the rapid economic development, the industrial structure of the country significantly changed. There was a clear decrease in the contribution share of the secondary sector, which declined from 57.4 to 34.4%. The contribution share of the tertiary sector gradually increased from 39.0 to 61.5%, surpassing that of the secondary sector in 2015. The contribution share of the primary sector remained relatively stable. The results indicate that the focus of China’s industrial structure development is gradually shifting to the tertiary sector.

The carbon emissions in China and their sectoral distribution from 2010 to 2018 are shown in Fig. 1b. In total, carbon emissions in China increased from 9.08 to 11.59 billion tons from 2010 to 2018, with an annual growth rate of 3.1%. The secondary sector was the main source of carbon emissions, and its carbon emissions as a percentage of the gross emissions varied from 71.5 to76.7%. The reason is that energy-intensive subsectors such as steel, concrete, and nonferrous metal account for a larger proportion in the secondary sector and produce large amounts of carbon emissions while consuming energy. The tertiary sector’s carbon emissions accounted for 12.3–15.5%. The primary sector emitted the least CO_2_, accounting for 1.6–1.8% of total carbon emissions.

Figure 1 c provides China’s total employment and its sectoral distribution from 2010 to 2018. Overall, the total employment increased from 761.05 million persons in 2010 to 775.86 million persons in 2018. The primary sector held the largest volume of employment in 2010, followed sequentially by the secondary and tertiary sectors. The primary sector’s employment proportion gradually declined, while that of the secondary and tertiary sectors gradually rose. In 2011, the tertiary sector’s proportion increased to 35.7%, surpassing that of the primary sector. In 2014, the secondary sector’s employment proportion reached 29.9%, also exceeding that of the primary sector. In 2018, the proportion employed in the primary sector fell to 26.1%, and with the advance of industrial restructuring, the proportion will continue to decline. Felipe et al. (2016) forecasted that the proportion of employment in the primary sector in China will fall to 18% in 2025 and further fall to 5% in 2044.

Furthermore, the changes in the proportions of the three economic sectors, particularly the transition of employment from the primary sector to the tertiary sector, show that China is undergoing a major de-industrialization process, similar to most developed and developing countries (Gozgor 2018). This phenomenon was also reflected in the secondary sectors’ continually declining contribution to the GDP. It is noteworthy that the share of CO_2_ emissions from the manufacturing sector slow decline, suggesting that de-industrialization moderating the CO_2_ emissions in China.

To analyze the quantitative relation between economic growth, employment, and CO_2_ emissions, this study calculated the carbon productivity and carbon employment rate of broad sectors. Carbon productivity refers to the ratio of the GDP to CO_2_ emissions in a certain period. It represents the efficiency of CO_2_ emissions during a period of economic growth and is an important indicator for measuring the low-carbon economy (Kaya and Yokobori 1999). By using the value added as a substitute variable for GDP, Long et al. (2016) extended this indicator to the industrial level. Based on the concept of carbon productivity, the ILO and CASS (2010) provided an additional definition of the carbon employment rate as the ratio of the employment and CO_2_ emissions, which describes the jobs created per unit of carbon emissions or the carbon emissions from increases in employment. Table 3 reports the results of carbon productivity and the carbon employment rate of broad sectors in 2010 and 2018.

**Table 3:** Carbon productivity and carbon employment rate of broad sectors

Sector	Carbon productivity (10^4^ yuan/t)		Carbon employment rate (persons/t)	
	2010	2018	2010	2018
Total	0.45	0.76	0.08	0.07
The primary sector	2.30	3.38	1.67	1.06
The secondary sector	0.28	0.42	0.03	0.03
Mining	0.61	1.20	0.05	0.04
Manufacturing	0.22	0.32	0.02	0.02
Production and supply of electricity, heat, gas, and water	0.37	0.53	0.02	0.02
Construction	3.45	5.18	0.61	0.60
The tertiary sector	1.63	2.55	0.24	0.20
Transport, storage and post	0.51	0.96	0.06	0.05
Wholesale and retail trades, hotels and catering services	1.99	2.87	0.31	0.29
Other Services	2.78	3.68	0.47	0.37

As shown in Table 3, owing to economic development, industrial restructuring, and improvements in energy efficiency, China’s carbon productivity increased from 0.45×10^4^ yuan/t in 2010 to 0.76×10^4^ yuan/t in 2018. The same situation presents at the sector level, where the carbon productivity of the primary, secondary, and tertiary economic sectors increased by 47%, 54%, and 57%, respectively. Among the broad sectors, the carbon productivities in mining, manufacturing, and the production and supply of electricity, heat, gas, and water increased significantly. One reason for this is that a series of key energy-saving technologies have been successfully applied during the 12th Five-Year Plan, such as the promote of new combustion technology and recovery of waste energy and heat. These technological innovations have made a great contribution to promoting the improvement of energy efficiency in relevant industries; thus, the output efficiency per unit of carbon emissions has increased. He et al. (2010) pointed out that the growth rate of carbon productivity could be used as an indicator to measure the effort of tackling global climate change within the framework of the sustainable development of an economy. The rapid increase in carbon productivity suggests that the pattern of economic development in China has been transforming to low-carbon development. However, the carbon productivity in China is currently relatively much lower than those in developed countries. To reach this advanced level, continuous efforts should be made to improve the carbon productivity. On the other hand, this means that there is huge potential in China in terms of combating global climate change (Long et al. 2016).

From a point of vertical comparison, evident differences can be observed at the sectoral level; that is, the carbon productivities of the primary and the tertiary sector are much higher than those of the secondary sector. The construction sector has the highest carbon productivity, whereas the manufacturing sector has the lowest. This finding is consistent with that of Yang et al. (2021), who indicated that, as the carbon productivity of most of manufacturing sectors are relatively low, equipment and technology in these sectors should be updated. Fan et al. (2021) also found that the improvement of carbon productivity in manufacturing sectors can significantly contribute toward achieving China’s goal of reducing their carbon emission intensity. It can thus be suggested that continuing to improve the carbon productivity of the secondary sector—particularly the manufacturing sectors—may be an effective approach to realizing the low-carbon transition. Several studies have shown that improving energy efficiency, technological progress, opening degree, and industrial scale structure have significantly positive effects on industrial carbon productivity; among these strategies, enhancing the energy efficiency is most effective (Long et al. 2016; Lu et al. 2018).

In contrast to the change in carbon productivity, the carbon employment rates showed a decreasing pattern. The total carbon employment rate decreased from 0.08 persons/t in 2010 to 0.07 persons/t in 2018, and the carbon employment rate of each sector remained relatively stable or decreased slightly. These decreases could be because the growth rate of carbon productivity was lower than that of labor productivity. Similar to the feature of carbon productivity in three economic sectors, it is apparent from Table 3 that the carbon employment rates of both the primary and the tertiary sector are much higher than that of the secondary sector. For example, in 2018, each ton of CO_2_ produced created 1.06 jobs in the primary sector and 0.20 jobs in the tertiary sector but only 0.03 jobs in the secondary sector. In other words, the environmental costs of creating the same jobs in the primary and tertiary sectors were lower than that in the secondary sector. Furthermore, in the broad sectors, the carbon employment rate in the construction sector was higher than those in other sectors, and fewer jobs per unit of emissions were generated in basic industry sectors, such as manufacturing, mining, and power generation and supply. For the tertiary sector, the carbon employment rate of consumer services is higher than that of production services. For example, a one-ton increase in CO_2_ emissions in 2018 produced only 0.05 jobs in the traffic, transport, storage, and post sector, whereas it created 0.29 jobs in the wholesale and retail trades, hotels, and catering sector and 0.37 jobs in other services.

The carbon productivity and carbon employment rate are both important indicators to measure the relative relationship between the negative costs (carbon emissions) and positive benefits (output or jobs) caused by economic activities. The higher the benefit per unit cost, the greater the economic operation efficiency, to some extent. From the perspective of the output benefit per unit of carbon emissions (carbon productivity), China’s economic efficiency has improved rapidly in recent years. However, in terms of jobs created per unit of carbon emissions, the decrease in the carbon employment rate shows that improved economic efficiency is not as positive as expected. This decrease also highlights the importance of paying more attention to employment during the economic transition. Taking the two indicators together, the primary and tertiary sectors have potential advantages for achieving low-carbon development and creating jobs in China. However, as an important pillar of the economy, the secondary sector—particularly the energy-intensive sectors—emits high emissions while providing material inputs for all of society (Long et al. 2016). Therefore, improving carbon productivity and carbon employment rate in the secondary sector is essential to promote economic efficiency and accelerate the low-carbon transition process in China.

### Economic and emission linkages analysis

To assess the sectoral economic and carbon emission linkage and compare different sectors’ impact on the economy and environment, the economic and carbon emission diffusion coefficients of 28 subsectors in China were calculated based on Eqs. (5)–(8). Table 4 shows the results in 2010–2018. In 2010, 16 subsectors had an economic diffusion coefficient greater than 1; these were mainly manufacturing, accounting for 67% of subsectors in the secondary sector. This somewhat demonstrates that manufacturing tends to be crucial as an engine of economic growth in China, which is in accordance with the results of Szirmai (2012). There were 11 subsectors with an economic diffusion coefficient greater than 1 in 2018. In terms of rankings, subsectors with the highest economic diffusion coefficients can be classified into three types. For example, S07, S08, and S09 are traditional light industries, whose products account for the majority of household consumption; S16, S17, and S18 are technology-intensive sectors with value-added products; S12, S13, and S15 are heavy chemical industries characterized by long industrial chains and close linkages. In addition, S25 showed a remarkable rise in the rankings, reaching the top in 2015 and showing its increasingly prominent economic performance.

**Table 4:** Economic diffusion coefficient and carbon emission diffusion coefficient of 28 subsectors in China

Sector	Economic diffusion coefficient (rankings)								Carbon emission diffusion coefficient (rankings)							
	2010		2012		2015		2018		2010		2012		2015		2018	
S01	0.87	(20)	0.92	(18)	0.92	(21)	0.91	(17)	0.55	(26)	0.60	(26)	0.66	(25)	0.65	(23)
S02	0.87	(21)	0.85	(22)	0.79	(25)	0.78	(23)	1.06	(12)	1.13	(9)	0.81	(18)	0.86	(17)
S03	0.74	(28)	0.76	(26)	0.62	(28)	0.61	(28)	0.79	(20)	0.89	(17)	0.75	(22)	0.69	(22)
S04	0.93	(18)	0.87	(21)	0.83	(23)	0.73	(26)	0.99	(14)	0.95	(15)	0.81	(19)	0.86	(16)
S05	0.98	(17)	0.93	(17)	1.03	(14)	0.98	(13)	1.09	(11)	1.13	(10)	1.12	(11)	1.19	(9)
S06	1.05	(13)	1.03	(14)	0.97	(18)	1.01	(11)	0.65	(25)	0.61	(25)	0.60	(26)	0.61	(25)
S07	1.15	(5)	1.18	(2)	1.10	(8)	1.19	(3)	0.98	(15)	1.03	(13)	0.96	(14)	1.20	(8)
S08	1.20	(1)	1.19	(1)	1.10	(7)	1.25	(1)	0.78	(21)	0.80	(22)	0.74	(23)	0.90	(15)
S09	1.16	(2)	1.13	(4)	1.10	(6)	1.09	(4)	0.88	(17)	0.82	(21)	0.81	(20)	0.77	(20)
S10	1.12	(7)	1.09	(9)	1.09	(11)	1.04	(8)	1.09	(9)	1.08	(12)	1.07	(12)	1.02	(12)
S11	0.78	(26)	0.75	(27)	0.73	(27)	0.67	(27)	0.84	(18)	0.85	(19)	0.84	(17)	0.91	(14)
S12	1.08	(11)	1.08	(10)	1.04	(13)	0.98	(12)	1.32	(6)	1.33	(7)	1.27	(7)	1.39	(6)
S13	1.09	(10)	1.05	(12)	1.09	(10)	1.06	(6)	3.08	(1)	3.32	(1)	2.85	(1)	3.11	(2)
S14	1.04	(15)	0.99	(16)	0.97	(19)	0.91	(16)	2.63	(2)	2.56	(2)	2.40	(2)	3.35	(1)
S15	1.14	(6)	1.11	(7)	1.13	(2)	1.07	(5)	1.58	(3)	1.54	(3)	1.54	(3)	1.80	(3)
S16	1.10	(8)	1.11	(8)	1.12	(4)	1.05	(7)	1.23	(7)	1.16	(8)	1.17	(9)	1.18	(10)
S17	1.15	(4)	1.13	(5)	1.10	(9)	1.03	(10)	1.03	(13)	1.03	(14)	0.99	(13)	0.97	(13)
S18	1.15	(3)	1.14	(3)	1.13	(3)	1.04	(9)	1.34	(5)	1.35	(6)	1.36	(6)	1.35	(7)
S19	1.05	(14)	1.07	(11)	1.03	(15)	0.88	(19)	0.74	(23)	0.72	(23)	0.71	(24)	0.60	(26)
S20	1.02	(16)	1.04	(13)	1.02	(16)	0.90	(18)	0.83	(19)	0.88	(18)	0.85	(16)	0.73	(21)
S21	0.86	(22)	0.80	(24)	1.05	(12)	0.77	(24)	0.75	(22)	0.85	(20)	1.14	(10)	0.79	(19)
S22	1.06	(12)	1.01	(15)	1.10	(5)	0.95	(14)	1.09	(10)	1.10	(11)	1.19	(8)	1.10	(11)
S23	0.84	(24)	0.77	(25)	0.95	(20)	0.75	(25)	0.71	(24)	0.64	(24)	0.78	(21)	0.64	(24)
S24	0.92	(19)	0.88	(19)	0.98	(17)	0.93	(15)	1.22	(8)	1.38	(5)	1.45	(5)	1.64	(4)
S25	1.09	(9)	1.12	(6)	1.16	(1)	1.21	(2)	1.44	(4)	1.54	(4)	1.49	(4)	1.60	(5)
S26	0.86	(23)	0.87	(20)	0.85	(22)	0.84	(21)	0.91	(16)	0.92	(16)	0.92	(15)	0.82	(18)
S27	0.75	(27)	0.72	(28)	0.73	(26)	0.81	(22)	0.47	(28)	0.44	(28)	0.47	(28)	0.49	(28)
S28	0.79	(25)	0.80	(23)	0.80	(24)	0.86	(20)	0.51	(27)	0.51	(27)	0.55	(27)	0.54	(27)

In 2010, 13 subsectors—all in the secondary sector—had carbon emission diffusion coefficients greater than 1; in contrast, there were 12 subsectors with carbon emission diffusion coefficient greater than 1 in 2018. Energy-intensive sectors with high carbon emissions, such as S13, S14, and S22, had the majority of top-ranking carbon emission diffusion coefficients. These sectors are upstream of the entire industrial chain, supplying energy and raw materials to other sectors. The carbon emission diffusion coefficients of light industries, such as S06 and S08, were less than 1 throughout the research period, indicating that their impacts on overall carbon emission are weak. It should be noted that the carbon emission diffusion coefficient ranking of S25 remained relatively high, given that the development of this sector significantly affects other related sectors’ carbon emissions. Therefore, we should not underestimate the role of the construction sector in reducing carbon emissions.

**Fig. 2:**
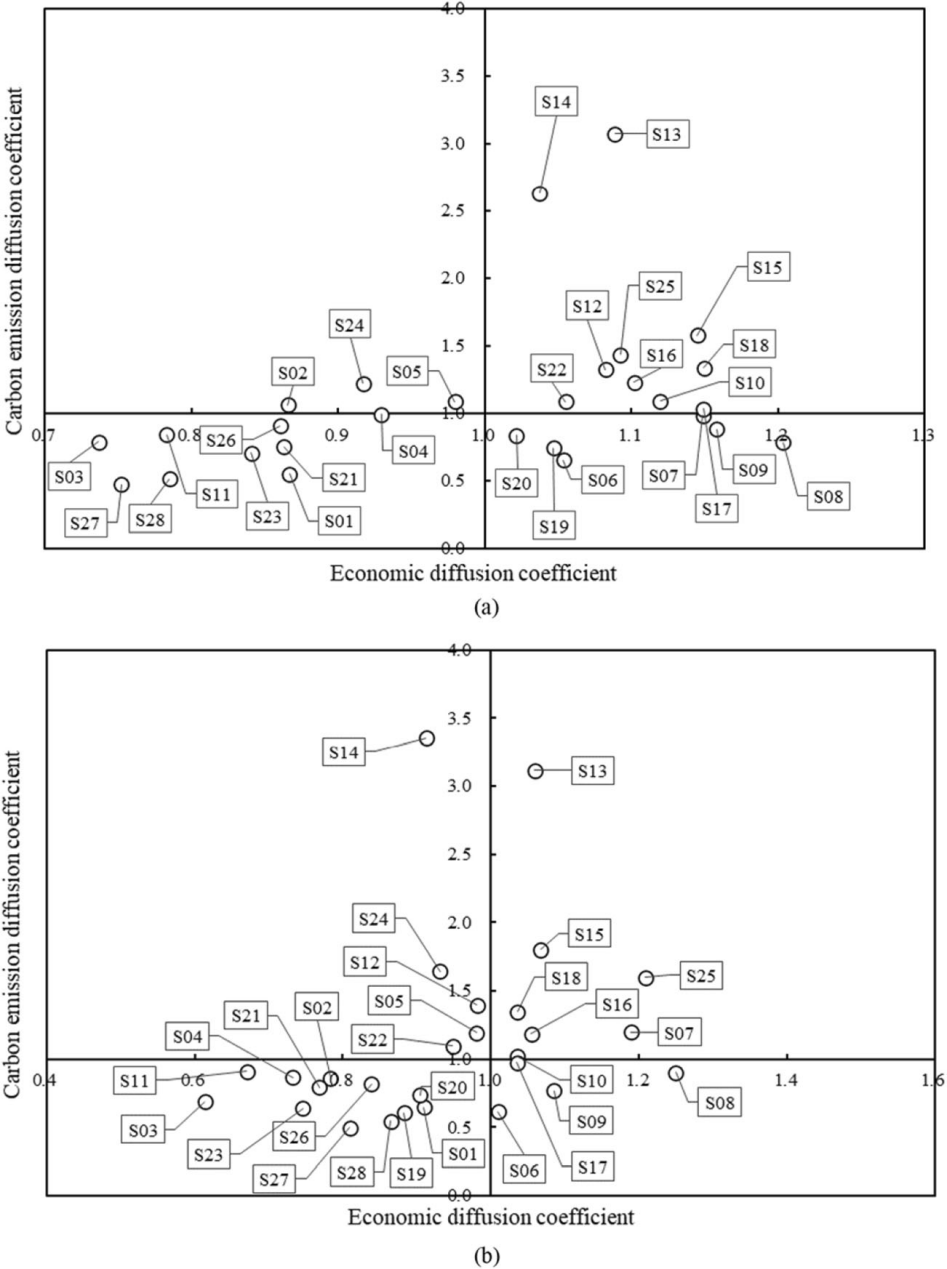
Clustering chart of 28 subsectors using the economic and carbon emission diffusion coefficients in 2010 (**a**) and 2018 (**b**)

It is evident that there is a conflict between economic and emissions performances in some sectors; reducing carbon emissions by output restriction will influence the economic income of some sectors and may hinder national economic growth to a certain degree. To maximize the prevention of economic losses due to carbon emissions reduction, it is necessary to conduct a combined analysis of economic and emission linkages. Thus, clustering analysis was carried out in this study. The sectors were clustered by the economic diffusion coefficient (*β*_*j*_) and carbon emission diffusion coefficient (*λ*_*j*_) calculated above. Consider the 2010 and 2018 data as an example. Figure 2a presents the clustered results in 2010. The majority of sectors were distributed in the first and third quadrants, containing ten and nine sectors, respectively; in contrast, there were six sectors in the fourth quadrant and only three sectors in the second quadrant. Sectors in the first quadrant, *β*_*j*_ > 1, *λ*_*j*_ > 1, are strong drivers of the national economy and carbon emissions. This study defines sectors in this quadrant, which are key sectors at the current, as being in sector group I. This group is most connected in terms of both economic and emission linkages; consequently, the total output and carbon emissions will change significantly if even relatively small changes are made to the sectors in this group. This type of sector mainly relates to the mining and processing of nonmetal ores and other ores, some heavy chemical sectors, the production and supply of electric and heat power, and construction. Sectors in the second quadrant, *β*_*j*_ < 1, *λ*_*j*_ > 1, have non-significant economic impacts despite significantly affecting carbon emissions. Sectors in this quadrant are defined as being in sector group II and should be appropriately restricted in the low-carbon transition process. This group contains only three sectors, S02, S05, and S24. Sectors in the third quadrant, *β*_*j*_ < 1, *λ*_*j*_ < 1, have non-significant impacts on both the economy and carbon emissions. Sectors in this quadrant are defined as being in sector group III and are typically either related to the fossil energy mining and processing sectors or labor-intensive sectors, such as agriculture and service. Sectors in the fourth quadrant, *β*_*j*_ > 1, *λ*_*j*_ < 1, are non-significant carbon emission sectors but have significant effects on economic growth. This study defines sectors in the fourth quadrant as being in sector group IV. These are mainly labor and technology-intensive sectors, which have priority for the low-carbon transition.

By comparing clusters for different periods, the evolution of sectoral linkages can be studied. Compared to 2010, there are evident changes in the overall clustering results in 2018 (see Fig. 2b). Among the sectors, S12, S14, and S22 changed from sector group I to group II, indicating that their ability to pull the economy decreased, while the impact on carbon emissions did not improve much. As the carbon emission diffusion coefficient decreased while the economic diffusion coefficient remained at a stable level, S02 changed from sector group II to group III, and S17 changed from sector group I to group IV. The carbon emission diffusion coefficient of S07 increased slightly, changing it from sector group IV to group I. As the economic diffusion coefficient decreased while the carbon emission coefficient remained stable, S19 and S20 changed from sector group IV to group III. These changes reflect the transition of industries to a certain extent, and the results somewhat shed light on the prospective direction of industrial restructuring. In summary, the secondary sector is key for the low-carbon transition, particularly the subsectors with long industrial chains, such as the heavy chemical and construction sectors. These sectors make outstanding contributions to economic development while generating the majority of carbon emissions within the sector and/or in related sectors. The upgrading of these sectors is essential to achieve the low-carbon transition in China at present.

This finding has important implications for emerging and developing economies such as China. Emerging economies have relatively high growth rates and have become a new engine driving global economic growth, as well as being the fastest growing group in terms of energy consumption and CO_2_ emissions. Given the current pressing climate change and global warming problems, both China and other emerging economies should choose between low carbon emissions and high economic growth. In general, upgrading the industrial structure benefits both the economic growth and carbon emission reductions. A combined analysis on economic and emission linkages may elucidate a sector’s potential for industrial structure upgrades. Identifying sectoral groups may help assist industrial policy, and focusing on key sectors is beneficial to accelerating the process of industrial restructuring. It may also provide a reference to help other emerging economies take action to reduce carbon emissions in a more sustainable way.

### Employment impacts analysis

The employment multipliers of various subsectors can be calculated using Eqs. (9)–(13). The employment multipliers (including total and subentry effects) and corresponding ratios of 28 subsectors in China are shown in Table 5. In general, for the majority of sectors, the indirect effects were greater than the induced and direct effects. The indirect effects were the main determiners of sectoral employment creation ability and accounted for about 40–70% of the total effect. Induced effects accounted for around 20% of the total effect, with the highest proportion reaching 40.9%. The agriculture and service sectors had relatively high direct effects, whereas other sectors’ direct effects were generally lower. Employment multipliers were generally lower in 2018 than in 2010. This implies that the impacts on employment arising from a unit of change in the final demand for a sector’s output generally suffered a decline. This is mainly caused by the decrease in the labor required to produce the same products due to advancements in production technology and increases in labor productivity. Table 5 shows the expansion of the proportion of indirect effects, reflecting the strengthened linkages between sectors that follow the optimization and upgrading of industrial structure. In contrast, there was a decrease in the proportions of direct and induced effects, indicating that the impacts of direct and induced effects on total employment effects are weakening.

**Table 5:** Employment multipliers and corresponding ratios of 28 subsectors in 2010 and 2018

Sector	2010							2018						
	Direct effects		Indirect effects		Induced effects		Total effects	Direct effects		Indirect effects		Induced effects		Total effects
S01	0.403	70.7%	0.113	19.8%	0.054	9.5%	0.570	0.225	71.9%	0.061	19.6%	0.027	8.5%	0.313
S02	0.039	31.9%	0.051	41.9%	0.032	26.2%	0.123	0.026	40.9%	0.028	42.9%	0.010	16.2%	0.064
S03	0.022	28.3%	0.033	43.6%	0.022	28.2%	0.077	0.009	29.1%	0.016	51.8%	0.006	19.2%	0.030
S04	0.032	28.8%	0.052	46.7%	0.027	24.5%	0.110	0.019	39.9%	0.022	44.7%	0.007	15.4%	0.048
S05	0.045	33.3%	0.059	44.1%	0.030	22.6%	0.134	0.031	38.5%	0.035	44.2%	0.014	17.3%	0.080
S06	0.019	5.7%	0.275	82.0%	0.042	12.4%	0.336	0.013	8.1%	0.129	81.8%	0.016	10.1%	0.157
S07	0.040	14.8%	0.192	71.0%	0.038	14.2%	0.270	0.018	12.8%	0.107	76.2%	0.015	10.9%	0.141
S08	0.042	16.9%	0.167	67.1%	0.040	16.1%	0.248	0.024	18.9%	0.087	69.1%	0.015	12.0%	0.126
S09	0.033	14.5%	0.155	69.0%	0.037	16.5%	0.224	0.019	18.0%	0.074	69.8%	0.013	12.2%	0.105
S10	0.030	17.2%	0.111	63.7%	0.033	19.1%	0.174	0.019	21.5%	0.058	65.5%	0.012	13.0%	0.089
S11	0.006	10.1%	0.038	62.2%	0.017	27.8%	0.061	0.003	13.1%	0.017	69.7%	0.004	17.2%	0.025
S12	0.020	14.2%	0.091	64.4%	0.030	21.4%	0.141	0.010	15.4%	0.047	70.6%	0.009	14.0%	0.066
S13	0.028	21.0%	0.074	55.7%	0.031	23.4%	0.133	0.021	27.0%	0.044	58.1%	0.011	14.9%	0.076
S14	0.015	14.8%	0.060	59.7%	0.026	25.5%	0.101	0.009	19.9%	0.030	64.2%	0.007	15.9%	0.047
S15	0.024	19.6%	0.070	56.5%	0.030	23.9%	0.124	0.019	27.5%	0.039	57.3%	0.010	15.2%	0.068
S16	0.023	19.4%	0.067	55.7%	0.030	24.9%	0.120	0.014	22.0%	0.039	62.1%	0.010	15.9%	0.063
S17	0.015	12.8%	0.071	61.3%	0.030	25.9%	0.116	0.007	13.0%	0.038	71.1%	0.009	15.8%	0.054
S18	0.019	15.8%	0.072	59.3%	0.030	24.9%	0.122	0.010	18.2%	0.038	66.4%	0.009	15.4%	0.058
S19	0.020	17.2%	0.065	56.7%	0.030	26.1%	0.115	0.011	22.4%	0.030	61.8%	0.008	15.8%	0.048
S20	0.028	23.1%	0.063	52.4%	0.029	24.5%	0.120	0.012	23.2%	0.032	60.7%	0.008	16.1%	0.052
S21	0.023	15.9%	0.097	67.4%	0.024	16.7%	0.145	0.020	34.3%	0.029	50.2%	0.009	15.5%	0.057
S22	0.013	12.5%	0.061	58.6%	0.030	28.9%	0.105	0.008	16.4%	0.033	66.0%	0.009	17.6%	0.050
S23	0.023	24.7%	0.045	48.2%	0.025	27.1%	0.093	0.012	29.1%	0.021	53.6%	0.007	17.3%	0.040
S24	0.055	38.8%	0.051	35.7%	0.036	25.4%	0.142	0.033	39.9%	0.037	44.3%	0.013	15.8%	0.083
S25	0.047	30.5%	0.072	46.6%	0.035	22.9%	0.155	0.031	30.5%	0.057	55.0%	0.015	14.6%	0.103
S26	0.047	34.8%	0.039	28.6%	0.050	36.6%	0.136	0.026	37.4%	0.031	45.7%	0.012	16.9%	0.069
S27	0.092	45.0%	0.046	22.4%	0.067	32.6%	0.205	0.057	49.3%	0.043	37.7%	0.015	13.1%	0.115
S28	0.098	53.3%	0.012	6.4%	0.074	40.2%	0.184	0.050	48.3%	0.037	36.1%	0.016	15.6%	0.103

S01 (agriculture, forestry, animal husbandry, and fishing) had the largest total effect, and its direct effect accounted for a large proportion. Its total effect in 2010 is 0.57, which decreased to 0.31 in 2018, and the direct effect accounted for 70.7% and 71.9% of the total effects, respectively. Agriculture has always assumed the function of the employment reservoir of China because of its strong pull factor on labor employment by generating direct employment compared to those of other sectors. However, the pull factor on effective employment of S01 may be overestimated; the proportion of agricultural labor calculated by official statistical data is higher than that in reality (Cai 2016), and there is a surplus labor problem in rural areas.

Among the subsectors in the secondary sector, the total effects of S06–S10 were relatively large and were dominated by indirect effects. These effects accounted for 63.7%–82.0% and 65.5%–81.8% of the total effects in 2010 and 2018, respectively. This shows that a unit increase in the final demand for output of these subsectors had a great impact on employment levels in their upstream and downstream sectors. These sectors were labor-intensive from the point of input of production factors, which can create a large number of employment opportunities in related sectors. In addition, the carbon emissions of these subsectors were generally comparatively lower than those of other subsectors based on the previous analysis. The result is consistent with the previous findings of Xue (2011), which revealed that improved energy saving and employment compatibility, as well as reduced emissions, can be achieved by the development of labor-intensive sectors. Among these subsectors, S07, S08, and S09 showed low carbon emissions and excellent employment pull factors on top of good economic performance, indicating that they are conducive to achieving a “multi-win” situation for economic development, carbon reduction, and employment level maintenance. As one of the pillar sectors in China, S25’s employment multiplier was also remarkable: its total effects were 0.15 and 0.10 in 2010 and 2018, respectively. This result is consistent with the “the 13th Five-Year Plan for the Development of the Construction Industry” (Ministry of Housing and Urban-Rural Development [MOHURD] 2017), in which the construction sector plays an important role in the absorption of rural labor transfer and the alleviation of social employment pressure. As mentioned in the previous section, most of the key subsectors belong to the secondary sector. Although these sectors are the main sources of carbon emissions, they also make outstanding contributions to economic development, and some of their employment multipliers, especially the indirect effects, are also relatively robust. Therefore, the output of these sectors cannot be blindly restrained for fear of negative impacts on the national economy and labor market.

The employment multipliers of S26–S28 (the tertiary sector) were dominated by their direct effects, although their induced effects were also remarkable. The tertiary sector is likened to a “sponge” that absorbs labor forces and provides a large number of jobs. Some service sectors, such as the traffic, transport, storage, and post and wholesale and retail trades, hotels, and catering sectors, have low education and professional skill requirements, thus playing a vital role in relieving employment pressure and accepting the transfer of rural surplus labor. Actively developing the service sectors is conducive to realizing low-carbon development while solving the problem of rural surplus labor transfer, thus creating a “double dividend” in terms of environmental protection and employment.

It is noteworthy that induced effects also play an important role. For the majority of sectors, induced effects accounted for around 15–30% of the total effects. This proportion was even more significant in the tertiary sector and was greater than 30% in 2010. This can be attributed to the fact that the induced effects are due to the changes in household consumption and income, and the tertiary sector covers a wide range of subsectors that are closely related to consumption. With the improvement of residents’ standard of living and the changes in consumption habits, residents’ consumption needs have changed from materials to services, and the proportion of service consumption in household spending has increased. The current data also highlight the importance of household consumption and income for employment. On the one hand, when studying employment impacts, neglecting the impact of household income and consumption changes on employment leads to the underestimation of the employment multipliers of various sectors to varying degrees; this problem becomes more obvious in the case of the service sector. Thus, it is necessary to pay attention to the induced effects and accurately measure them. On the other hand, vigorously expanding household consumption and increasing household income would be effective tools for stabilizing employment.

China’s economic structure has undergone evident changes, and consumption has become the primary driving force for economic growth. According to the data from the National Bureau of Statistics, China’s total retail sales of consumer goods reached 41.2 trillion yuan in 2019, an annual increase of 8%, and the contribution of consumer spending to GDP growth reached 57.8%. Meanwhile, household spending mainly reacts to the service sector. With the continuous improvement of people’s standards of living, the proportion of services such as education, culture, and entertainment in household consumption expenditures has continued to increase. Boosting consumption will undoubtedly promote the development of the service sector, especially the consumer service sectors, which are characterized by low emissions. It is possible, therefore, that actively expanding consumption and increasing income could be an option to achieve the multi-win situation of economic growth, job creation, and carbon emissions reduction.

**Fig. 3:**
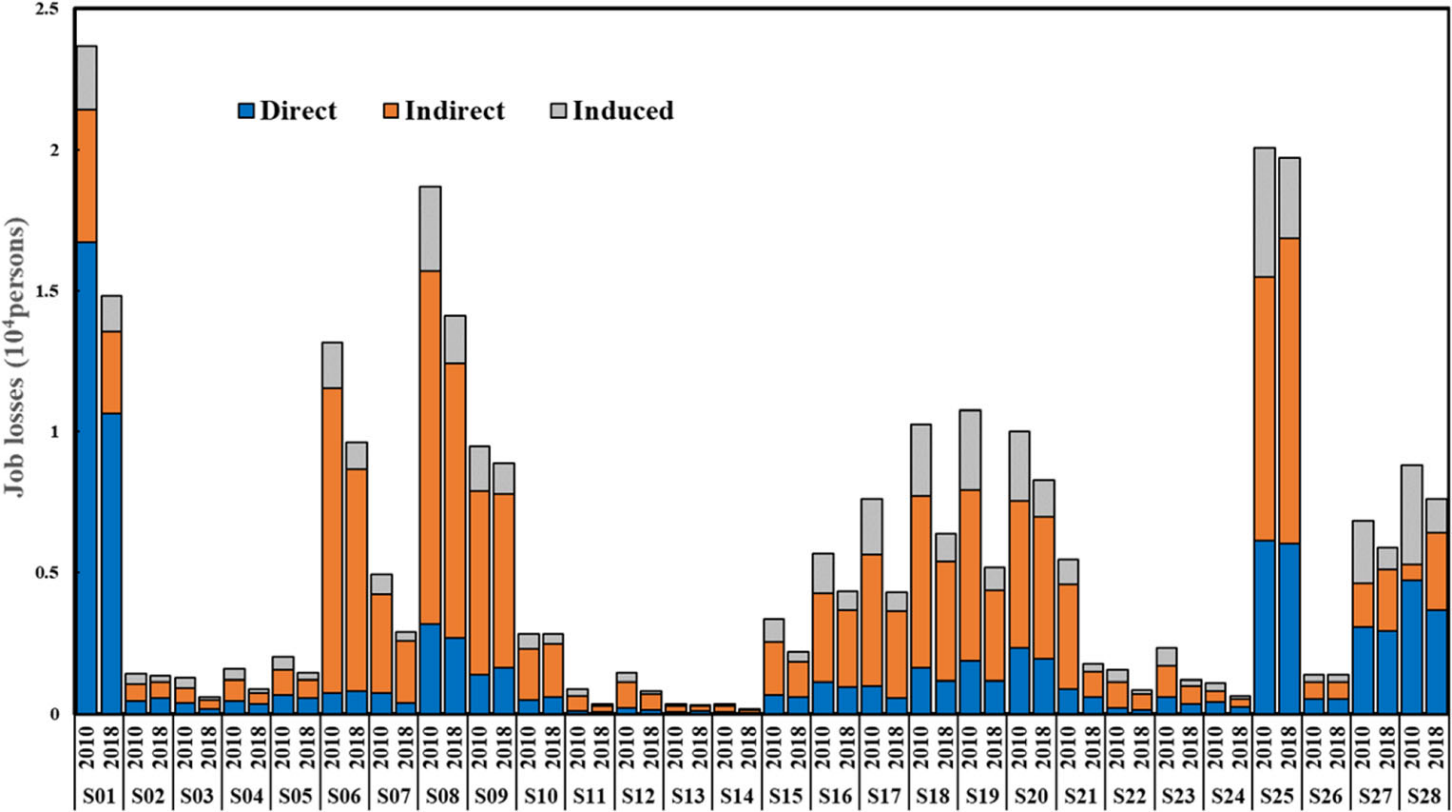
Job losses per unit of carbon emissions reduction in each subsector in 2010 and 2018

The measurement of job losses provides an intuitive way to assess the employment impacts of carbon emissions reduction. According to Eq. (14), by multiplying the employment multipliers of the above sectors by the corresponding output per unit of CO_2_ emissions, the job losses per unit of carbon emissions reduction is obtained. The results from 2010 and 2018 are shown in Fig. 3. It is notable that the job losses per unit of carbon emissions reduction in the primary sector (S01) decreased from 2.37 to 1.48 between 2010 and 2018, a decrease of more than 37%. Among the subsectors in the secondary sector (S02–S25), S09 and S10 increased, while other sectors decreased in terms of the job losses per unit of carbon emissions reduction. The job losses per unit of carbon emissions reduction in the tertiary sector (S26–S28) decreased slightly between 2010 and 2018. In 2010, S01 would cause the largest job losses if carbon emissions were reduced one unit; this was surpassed by that of S25 in 2018. The job losses per unit of carbon emissions reduction in some sectors (S06, S08, S09, S18, S19, S20, S27, and S28) were also considerable. The proportions of direct job losses in S01 and S26–S28, as shown in Fig. 3, were relatively high, highlighting that carbon emissions reductions in these sectors significantly affect intra-sector employment. By contrast, equivalent carbon emissions reduction in the secondary sector indirectly causes more job losses in other sectors than in itself. This effect is particularly prominent in labor-intensive subsectors S06–S10, indicating that these subsectors have important implications for employment in their upstream and downstream sectors. By comparing 2018 from 2010, it can be observed that the tertiary sector’s direct and induced job losses were reduced, while the indirect job losses rose, suggesting that carbon emissions reduction by limiting the development of the service sector would noticeably result in noticeable unemployment in other sectors.

In summary, one of the most significant findings is that indirect job losses account for a large proportion of the total job losses for most sectors’ carbon emissions reduction, and the induced job losses also cannot be neglected along with the direct job losses. In other words, policy designed to reduce carbon emissions should consider the employment impacts not only on the sector itself but also on the entire industry chain. If the employment impacts cannot be comprehensively evaluated during the industrial restructuring and low-carbon transition processes, unreasonable policy measures may be adopted to guide industrial restructuring and reduce carbon emissions. These policies could easily lead to the shedding of jobs by various sectors, which will eventually bring about high levels of unemployment and could even threaten social stability.

The present results are significant in at least two major respects. On the one hand, similar to the relationship between low carbon emissions and high economic growth, it can be inferred that China also needs to balance the relationship between low carbon emissions and job creation. The job losses per unit of carbon emissions reduction is a useful indicator to quantify the employment impacts of carbon emissions reduction, which may contribute to reducing the shocks to labor employment caused by carbon emissions reduction policies. On the other hand, this study raises the possibility that tackling unemployment problems by promoting indirect and induced employment may be more effective than directly increasing jobs. Strengthening sectoral linkages will help promote indirect employment, and expanding household consumption and increasing household income will contribute in promoting induced employment.

Prior studies have noted the importance of the household in driving economic growth and shaping energy use and environmental emissions in China. This study found that the household plays an indispensable role in stabilizing employment. After China entered a new normal in terms of economic development, the effect of domestic consumption (especially household consumption) on driving growth is more significant. Household consumption is expected to become a stronger driver of China’s economic growth in the foreseeable future. However, as consistent incomes and urbanization increase, the energy demands and carbon emissions of households will probably increase as well. Changing consumer behavior can often be regarded as an effective measure for reducing household-related energy use and emissions. Dai et al. (2012) found that demand-side initiatives, aiming to change household behavior toward more sustainable and low-carbon consumption, will increase the proportion of service and decrease that of industry. Therefore, a large amount of energy and carbon emissions could be saved and reduced, respectively. In summary, these findings suggest that expanding consumption (especially low carbon consumption) while increasing household income may help achieve sustainable environmental, economic, and social development.

## Conclusions and policy implications

Based on the semi-closed input–output model with partially endogenized consumption, this study first identified key sectors in terms of the economic and carbon emissions linkages; then quantified the direct, indirect, and induced employment multipliers and further measured the job losses per carbon emissions reduction; and, finally, industrial policy implications were presented based on the comprehensive assessments of the carbon emissions reductions, economic growth, and employment impacts.

In principle, holding everything else constant, carbon emissions can be reduced by policies that limit the output of high-emission sectors while expanding the output of low-emission sectors. However, in practice, carbon emission is not the only policy concern. An intelligently designed industrial policy could therefore influence a country’s development, so that it generates more and better domestic employment, emit less CO_2_ and other greenhouse gasses, and increases the overall productivity and competitiveness of its national economy. Such a policy would have to focus more on linkage and impact analysis.

The linkage analysis conducted in this study may provide useful information for identifying the direction of industrial restructuring for a low-carbon transition. By analyzing the intersectoral economic and emission linkages, this study found that, in China, the secondary sector is key for the low-carbon transition; particularly of note are most heavy chemical and construction sectors, which have a notable impact on the emissions of upstream and downstream sectors (owing to their long industrial chains) and make outstanding contributions to economic development. Pushing for progress in energy conservation and emissions reductions in these sectors is the key to realizing the low-carbon transition and sustainable development of China. In addition, some labor-intensive and technology-intensive sectors, which contribute to economic growth as well as low carbon emissions, are significant.

As one of the most important social impacts, employment impacts deserve more attention, and a full analysis of these impacts may contribute to reducing the uncertainties of the true effects of the low-carbon transition. Whether in terms of employment multipliers or job losses per carbon emissions reduction, the results show that the indirect effects play a major role in employment impacts. With the improvement of the industrial system and the extension of the industrial chain, the indirect employment brought about by the sectoral linkage will increase significantly. Induced effects are also important in employment impacts analysis, accounting for around 20% of the total effects. As the average income increases and consumption levels continue to rise, and the capacity for job creation in the service sector continues to improve, the positive impacts of household consumption and income on employment may become more prominent. The results suggest that when designing policies to reduce carbon emissions, more attention should be paid to the indirect employment impacts related to the entire industry chain, the induced impacts resulting from changes in household consumption and income, and the direct employment impacts on the industry itself.

In conclusion, in terms of production sectors, for sectors with non-significant economic impact but significantly impacts on carbon emissions, and whose per unit of carbon emissions reduction would also generally lead to few job losses, properly limiting their output may cause less negative effects. However, for key sectors with strong pull factors on the economy and high job losses per unit of carbon emissions reduction, such as the mining and processing of nonmetal ores and other ores, heavy chemical, production and supply of electric and heat power, and construction sectors, policies would have to focus more on industrial upgrading than on output restriction. Furthermore, some labor-intensive sectors, particularly the service sector, are conducive to achieving a “multi-win” situation in terms of economic development, carbon emissions reductions, and employment stability; thus, production in these sectors should be encouraged. In terms of households, the evidence from this study suggests that expanding consumption (especially low carbon consumption) and increasing household income are beneficial to achieving the sustainable development of economic, environmental, and social systems.

Based on the above analysis, the following policy recommendations are proposed.

First, policy-making should avoid one-size-fits-all tactics; identifying sectoral groups is very useful for assisting industrial policy, and focusing on key sectors is beneficial accelerating the process of low-carbon development. Industrial restructuring urges a reasonable distinguishing of sectoral groups; formulating different industrial policies according to differences in the sectoral groups while seeking a balanced point of cooperation can help achieve a multi-win situation for the environmental, economic, and social systems. For China’s key sectors, as defined by economic and carbon linkage characteristics, it is suggested to phase out backward production capacity, upgrade industry through reasonable planning, increase management efficiency in enterprises, and promote reductions in energy intensity and increases in carbon productivity by enhancing energy efficiency and technological innovation.

Second, employment policies should be compatible with industrial policies and avoid possible shock to the labor market resulting from the industrial restructuring. An accurate quantification of employment impacts can provide useful information for the government to formulate scientific policies. According to the results of this study, strengthening industrial chain cooperation and realizing industrial inter-connected development will be an effective measure to promote employment. As household consumption and income have evident impacts on employment, vigorously expanding consumer spending and increasing household income will serve as an employment stabilizer. Further, the service sector plays an important role in generating jobs. Actively developing the service sector is conducive to realizing industrial low-carbon development while solving the problem of rural surplus labor transfer in China.

Although this study is from the perspective of industrial restructuring, there is no doubt that the development and utilization of renewable energy also play an active role in diminishing energy dependence and reducing carbon emissions. Simultaneously, most studies have a positive attitude with regard to the renewable energy sector providing opportunities to create new jobs. Therefore, great efforts should be made to promote the development of new strategic low-carbon sectors, such as new energy and renewable energy, and to adjust and improve the energy mix while accelerating the restructuring and upgrading of traditional industries.

This study has some limitations. First, the scope of this study is limited to evaluate the actual impact of China’s economic activities on its domestic economy, environment, and employment; the input–output framework of this study does not cover the international input use. Trade is very important for China and caused the transfer of both emissions and labor. Consequently, the net impact after considering the import goods factor is still uncertain. The role of trade in shaping resource use and environmental emissions in China and the “trade in employment” based on the cross-border migration of new job opportunities is worth further analysis. Thus, we plan a further study on trade embodied carbon emissions and embodied labor. Second, although the diffusion coefficient is an important parameter to let normalized backward linkages be the measures, higher diffusion coefficients do not show how a sector has linked the separate sectors. Therefore, deepen sectoral linkages analysis combined with evaluation of the structure of supply chains and industrial chains is meaningful and a promising area for future research.

## Appendix A

The appendix presents the modeling process of the semi-closed input–output model with partially endogenized consumption.

The most important step in modeling is to divide the household consumption into endogenous and exogenous consumption by using the decomposition formulas. According to the relevant consumption theory (Friedman 1957; Modigliani 1986), in addition to the current income, factors affecting household consumption may include wealth, future income, consumption habits, interest rates, consumer preferences, product prices, demographic structure, and social factors. Considering the above factors, the consumption decomposition formula is expressed as follows:

15 $$ \left\{\begin{array}{l}{c}_{it}={c}_{it}^{en}+{c}_{it}^{ex}\\ {}{c}_{it}^{en}={\alpha}_{it}\left(\tilde{e},r,d,p,\lambda \dots \right){x}_{\left(n+1\right)t}\\ {}{c}_{it}^{ex}={\beta}_i{c}_{i\left(t-1\right)}+{\varepsilon}_{it}\end{array}\right.. $$

The explanations of each symbol in the decomposition formula are shown in Table 6. The estimation of the endogenous consumption coefficient *α*_*it*_ in Eq. (15) is key to decomposing consumption. The rational expectation theory assumes that the endogenous consumption coefficient follows the random walk process (Mankiw 2010):

16 $$ {\alpha}_{it}={\alpha}_{i\left(t-1\right)}+{\mu}_{it},{\mu}_{it}\sim NIID\left(0,{\sigma}_{\mu_{it}}^2\right). $$

**Table 6:** Explanations of symbols in decomposition formulas

Symbols	Explanations
*c*_*it*_	The total household consumption of products *i* in the period *t*
${c}_{it}^{en}$	The endogenous consumption of products *i* in the period *t*
${c}_{it}^{ex}$	The exogenous consumption of products *i* in the period *t*
*c*_*i*(*t* − 1)_	The total household consumption of products *i* in the period *t* − 1
*x*_(*n* + 1)*t*_	The total household income in the period *t*
*α*_*it*_	The endogenous consumption coefficient of products *i*
$\tilde{e}$	The expected future incomes
*r*	The interest rate
*d*	The demographic characteristics
*p*	The commodity price index
*λ*	The consumer preference parameter
*β*_*i*_	A coefficient determines the effect of the previous consumption peak on the current household consumption.
*ε*_*it*_	A random error term

The estimation process of endogenous consumption coefficient adopts the time-varying parameter model, maximum likelihood estimation, and Kalman filtering algorithm (Hamilton 1994; Havey 1987). The forms of the measurement equation and transition equation are expressed as follows:

17 $$ \left\{\begin{array}{l}{c}_{it}={\alpha}_{it}{x}_{\left(n+1\right)t}+{\beta}_i{c}_{i\left(t-1\right)}+{\varepsilon}_{it}\\ {}{\alpha}_{it}={\alpha}_{it-1}+{\mu}_{it}\end{array}\right.. $$

where *ε*_*it*_ and *μ*_*it*_ are independent disturbance vectors.

Given that consumption preferences are aimed at the types of consumption activities (such as food, clothing, and housing) rather than the types of products, the endogenous consumption coefficient of the input–output sectors cannot be directly estimated; thus, the endogenous consumption coefficient of consumption activities is first estimated. According to Eq. (17), the endogenous consumption coefficient vector of consumption activities ***c**** can be estimated. Table 7 presents the estimation results of the eight consumption categories in China. To obtain the endogenous consumption coefficient of input–output sectors, a bridge matrix is needed here to link the consumption activities to input–output sectors:

18 $$ \mathbf{c}={\mathbf{Bc}}^{\ast}. $$

where **c** refers to the endogenous consumption coefficients vector of input–output sectors and **c**^**∗**^ refers to the endogenous consumption coefficients vector of consumption activities. ***B*** is a bridge matrix and its form is expressed in Eq. (19):

19 $$ \mathbf{B}=\left[\begin{array}{cccc}{b}_{11}& {b}_{12}& \cdots & {b}_{1m}\\ {}{b}_{21}& {b}_{22}& \cdots & {b}_{2m}\\ {}\vdots & \vdots & \vdots & \vdots \\ {}{b}_{n1}& {b}_{n2}& \cdots & {b}_{nm}\end{array}\right], $$

**Table 7:** Endogenous consumption coefficients of the eight consumption categories in China

Year	Endogenous consumption coefficients							
	M1	M2	M3	M4	M5	M6	M7	M8
2010	0.092	0.058	0.079	0.018	0.053	0.076	0.030	0.018
2011	0.090	0.062	0.074	0.019	0.050	0.079	0.033	0.019
2012	0.088	0.060	0.071	0.017	0.055	0.079	0.032	0.020
2013	0.065	0.053	0.165	0.018	0.051	0.086	0.037	0.014
2014	0.080	0.052	0.159	0.019	0.060	0.087	0.039	0.015
2015	0.078	0.050	0.158	0.018	0.063	0.090	0.039	0.015
2016	0.073	0.048	0.161	0.019	0.068	0.093	0.040	0.015
2017	0.067	0.047	0.164	0.021	0.072	0.096	0.040	0.014
2018	0.062	0.045	0.167	0.022	0.077	0.099	0.041	0.013

M1, food; M2, clothing, residence; M3, household facilities; M4, articles and services; M5, transport and communication; M6, education, cultural, and recreation services; M7, health care and medical services; M8, miscellaneous goods and services

The element *b*_*ij*_ is the value of the product *i* required for the consumption activity *j* per unit quantity. The estimating process of the bridge matrix ***B*** mainly includes the following: (1) adjusting the column of household consumption in the input–output table to make aggregated household consumption of *n* sectors consistent with the sum of *m* consumption activities, (2) constructing a consumption flow matrix ***R*** and assigning initial values by dividing *m* consumption activities into *n* input–output sectors, (3) using the RAS method to balance the matrix ***R***, and (4) dividing each element of the balanced matrix ***R*** by the sum of its corresponding column to obtain the bridge matrix ***B****.* On this basis, the endogenous consumption coefficient of input–output sectors can be obtained using Eq. (18). Table 8 presents the estimation results of the 28 subsectors in China.

**Table 8:** Endogenous consumption coefficients of 28 subsectors in China

Sector	2010	2011	2012	2013	2014	2015	2016	2017	2018
S01	0.023	0.025	0.027	0.021	0.023	0.020	0.020	0.021	0.022
S02	0.001	0.001	0.001	0.001	0.001	0.001	0.001	0.001	0.000
S03	0.000	0.000	0.000	0.000	0.000	0.000	0.000	0.000	0.000
S04	0.000	0.000	0.000	0.000	0.000	0.000	0.000	0.000	0.000
S05	0.000	0.000	0.000	0.000	0.000	0.000	0.000	0.000	0.000
S06	0.062	0.066	0.066	0.062	0.068	0.064	0.066	0.066	0.064
S07	0.002	0.002	0.003	0.003	0.004	0.004	0.004	0.004	0.004
S08	0.036	0.036	0.033	0.036	0.038	0.038	0.040	0.039	0.038
S09	0.002	0.002	0.002	0.002	0.003	0.003	0.003	0.003	0.003
S10	0.004	0.006	0.008	0.012	0.016	0.020	0.019	0.018	0.020
S11	0.004	0.004	0.004	0.005	0.005	0.007	0.008	0.007	0.007
S12	0.010	0.012	0.013	0.015	0.018	0.020	0.021	0.020	0.020
S13	0.002	0.002	0.002	0.002	0.002	0.003	0.003	0.003	0.003
S14	0.000	0.000	0.000	0.000	0.000	0.000	0.000	0.000	0.000
S15	0.001	0.001	0.001	0.001	0.001	0.001	0.001	0.001	0.001
S16	0.000	0.001	0.001	0.001	0.001	0.001	0.001	0.001	0.001
S17	0.013	0.014	0.016	0.018	0.022	0.025	0.025	0.024	0.026
S18	0.009	0.009	0.008	0.009	0.010	0.010	0.011	0.010	0.011
S19	0.009	0.009	0.009	0.011	0.013	0.014	0.014	0.014	0.019
S20	0.001	0.001	0.000	0.001	0.001	0.001	0.001	0.001	0.002
S21	0.008	0.004	0.001	0.001	0.001	0.001	0.000	0.001	0.001
S22	0.015	0.014	0.012	0.013	0.013	0.013	0.014	0.013	0.013
S23	0.005	0.005	0.005	0.006	0.007	0.007	0.008	0.008	0.008
S24	0.005	0.004	0.003	0.003	0.003	0.004	0.004	0.004	0.004
S25	0.007	0.003	0.000	0.000	0.000	0.000	0.000	0.000	0.000
S26	0.031	0.036	0.040	0.044	0.043	0.041	0.045	0.043	0.048
S27	0.068	0.075	0.079	0.089	0.083	0.078	0.083	0.081	0.082
S28	0.083	0.071	0.063	0.105	0.103	0.097	0.083	0.091	0.090

After obtaining the endogenous consumption coefficients of 28 subsectors corresponding to the input–output tables, it is easy to decompose the household consumption into two parts using Eq. (15).

## Footnotes

1 In some analytical frameworks (Cloutier and Thomasin 1994; Miller and Blair 2009), the interactions between the household row and household column are positive values. In these frameworks, this value denoted households’ purchases of labor services. This value is considered zero in this study because there is a specific sector called household service and other social services in the input–output tables of China, and households’ purchases of labor services are captured by labor compensation in this sector.
